# Heart disease risk factors detection from electronic health records using advanced NLP and deep learning techniques

**DOI:** 10.1038/s41598-023-34294-6

**Published:** 2023-05-03

**Authors:** Essam H. Houssein, Rehab E. Mohamed, Abdelmgeid A. Ali

**Affiliations:** Faculty of Computers and Information, Minia University, Minia, Egypt

**Keywords:** Machine learning, Computer science

## Abstract

Heart disease remains the major cause of death, despite recent improvements in prediction and prevention. Risk factor identification is the main step in diagnosing and preventing heart disease. Automatically detecting risk factors for heart disease in clinical notes can help with disease progression modeling and clinical decision-making. Many studies have attempted to detect risk factors for heart disease, but none have identified all risk factors. These studies have proposed hybrid systems that combine knowledge-driven and data-driven techniques, based on dictionaries, rules, and machine learning methods that require significant human effort. The National Center for Informatics for Integrating Biology and Beyond (i2b2) proposed a clinical natural language processing (NLP) challenge in 2014, with a track (track2) focused on detecting risk factors for heart disease risk factors in clinical notes over time. Clinical narratives provide a wealth of information that can be extracted using NLP and Deep Learning techniques. The objective of this paper is to improve on previous work in this area as part of the 2014 i2b2 challenge by identifying tags and attributes relevant to disease diagnosis, risk factors, and medications by providing advanced techniques of using stacked word embeddings. The i2b2 heart disease risk factors challenge dataset has shown significant improvement by using the approach of stacking embeddings, which combines various embeddings. Our model achieved an F1 score of 93.66% by using BERT and character embeddings (CHARACTER-BERT Embedding) stacking. The proposed model has significant results compared to all other models and systems that we developed for the 2014 i2b2 challenge.

## Introduction

Heart disease is the leading cause of death in the United States, the UK, and worldwide. It causes more than 73,000 and 600,000 deaths per year in the UK and the US, respectively^1,2^. Heart disease caused the death of about 1 in 6 men and 1 in 10 women. Heart disease has a number of common forms such as Coronary Artery Disease (CAD). According to the World Health Organization, risk factors of a specific disease are any attributes that raise the probability that a person may get that disease^3^. There are several risk factors for CAD and heart disease such as Diabetes, CAD, Hyperlipidemia, Hypertension, Smoking, Family history of CAD, Obesity, and Medications associated with the mentioned chronic diseases^4–6^. Each heart risk factor should be specified with indicator and time attributes except for a family history of CAD and smoking status. Each indicator attribute reflects the implications of the risk factor in the clinical text. It is essential to detect risk factors mentioned in narrative clinical notes for heart disease prediction and prevention which is considered an important challenge.

Manually detecting heart disease risk factors from several forms of clinical notes is excessively expensive, time-consuming, and error-prone. Therefore, for efficient identification of heart disease risk factors, it is required to apply a model that is fine-tuned to the text structure, the clinical note contents, and the project requirements^7, 8^.

Electronic health records (EHRs) have been proved to be a promising path for advancing clinical research in recent years^9–11^. Although EHRs hold structured data such as diagnosis codes, prescriptions, and laboratory test results, a large portion of clinical notes are still in narrative text format, primarily in clinical notes from primary care patients. The narrative form of clinical notes is considered a major challenge facing clinical research applications^12^.

NLP techniques have been applied to convert narrative clinical notes into a structured format that will be effectively used in clinical research^13–15^. Furthermore, several studies have demonstrated the significant impact of NLP, machine learning, and deep learning techniques for disease identification using clinical notes, which are discussed as related works in this paper. Thus, our goal is to develop a model that can detect and predict the progression of heart disease and CAD from clinical notes. The prediction of heart disease risk factor using clinical and statistical approaches has attracted a lot of attention over the past ten years^16–20^ because this process is very complex. Several techniques have been applied to clinical concept extraction such as simple pattern matching, statistical systems, and machine learning. Although these techniques have achieved better results, it is difficult to apply such statistical models to analyze the EHR data due to the time-consuming process of processing large amounts of data, their usage of several statistical and structural assumptions, and custom features/markers^21, 22^.

Deep learning, a branch of machine learning that has made significant development recently, is used to create significantly improved NLP models^23^. DL approaches have lately made substantial progress in a variety of domains through the effective collection of long-range data relationships and the deep hierarchical creation of feature sets^24^. Due to the growing development of DL methods and the growing number of patient records that provide improved results and require less time-consuming preprocessing and feature extraction compared to conventional methods, there is an increase in research studies that apply DL techniques to EHR data for Clinical tasks^25, 26^.

Clinical text datasets with annotations are rare and small in size. This made it difficult to apply modern supervised DL techniques. To overcome this issue, clinical information extraction techniques based on transfer learning using pre-trained language models have recently become increasingly popular^27–33^.

Several studies have pre-trained these models on English biomedical and clinical notes^28, 29, 34, 35^ and fine-tuned them on several clinical downstream tasks^27, 30^. These models have widely applied the architecture of bidirectional encoder representations from transformers (BERTs).

This motivated the significance of the evaluation of pretraining and fine-tuning BERT on The i2b2 heart disease risk factors challenge dataset from the heart disease domain to highlight the efficiency of deep-learning-based NLP techniques for clinical information extraction tasks.

This paper proposed an advanced technique of using stacked embeddings to improve the previous research on the i2b2 2014 challenge. The i2b2 heart disease risk factors challenge dataset has shown significant improvement for stacking embeddings, which is conceptually a means to integrate several embeddings. We have achieved an F1-score of 93.66% on the test set by stacking BERT and character embeddings (CHARACTER-BERT Embedding). The main objective is to identify the risk factor indicators included in each document, as well as the temporal features related to the document creation time (DCT) using the data set from the i2b2/UTHealth shared task^10^.

Among all the models we have created as a part of this proposed model, this has demonstrated the best results. This is a promising result for our model’s potential to advance research beyond the current benchmark for DL models developed for this shared task^7^, which reported an F1 score of 90.81% using BLSTM and the most successful system^36^ of the i2b2/UTHealth 2014 challenge, which reported an F1 score of 92.76%. Additionally, our method focuses on how contextual embeddings help to further improve the effectiveness of NLP and DL. This research is a step toward a system that can outperform human annotators and surpass the current state-of-the-art results with minimal feature engineering.

In summary, the main objectives of this study are as follows:

- Developing a model that detects heart disease risk factors using stacked embedding algorithms by stacking BERT and CHARACTER-BERT Embedding. Furthermore, the utilization of DL approach (RNN) to extract risk factor indicators from the shared task dataset.
- Improve on work that has already been done in this space as part of the i2b2 2014 challenge.
- The proposed model achieved superior results compared to state-of-the-art models from the 2014 i2b2/UTHealth shared task.
- Various metrics are provided to assess the performance of the proposed model.

The remainder of the paper is organized as follows, “Related works” section, provides a detailed overview of the related work, highlighting several recent related works. The basic description of the dataset, the task, and clinical word embeddings are introduced in “Material and methods” section. “The proposed heart disease risk factors detection model” section, presents the proposed model steps by explaining preprocessing steps, describing the pre-trained word embeddings, and stacked word embeddings. “Discussion” section, shows the evaluation and the results of the proposed model. Finally, “Conclusion and future work” section, discusses the conclusion and future works.

## Related work

### Clinical information extraction using deep learning

Medical research highly depends on text-based patient medical records. Recent studies have concentrated on applying DL to extract relevant clinical information from EHRs. One of the most significant NLP task is the extraction of clinical information from unstructured clinical records to support decision-making or provide structured representation of clinical notes. The goal of this concept extraction challenge can be described as a sequence labeling problem, to assign a clinically relevant tag to each word in an EHR^37^. Different deep learning architectures based on recurrent networks, such as GRUs, LSTMs, and BLSTMs, were examined by^37, 38^. All the RNN versions outperformed the conditional random field (CRF) baselines, which were previously thought to be the most advanced technique for information extraction in general. Clinical event sequencing can be used to analyze disease progress and predict oncoming disease states as patient EHRs change over time^39^. Because of its temporality, it is necessary to give each extracted medical concept a sense of time^40^ proposed a solution for much more complex issues by using A typical RNN initialized with word2vec^41^ vectors and DeepDive^42^ for developing associations and predictions. While^43^ and^44^ also used word embedding vectors, they extracted the temporal attributes using CNNs. While these methods are not modern, they generated the best results in extracting temporal event. Additionally, each subtask requires a different model and some manual engineering, such as when extracting concepts and temporal attributes^45–47^. There is an important issue that none of the current systems have ever attempted to use a single, universe model that automatically identifies the temporal attributes of those factors based on their contexts and combines them into the feature learning process, which can be used to extract both medical factors and temporal attributes simultaneously.

### The i2b2/UTHealth shared task

The i2b2 has released several NLP shared challenging tasks that focused on identifying risk factors for heart disease in clinical notes as listed in Table 1. For example, the 2009 i2b2 shared task focused on detecting all medications mentioned in a dataset of 251 clinical notes and all relevant information such as reasons, frequencies, dosages, durations, modes, and whether the information was written in a narrative note or not^48^. The 2006 i2b2 shared task focused on classifying the smoking status of the patient into five classes: Past Smoker, Current Smoker, Smoker, Non-Smoker, and Unknown^49^. Similarly, the 2008 i2b2 shared task focused on classifying obesity and comorbidities status of the patient into four categories^50^.

There are three tracks participated in the 2010 i2b2/VA shared task^51^:

1. Clinical Concept extraction task, in which systems needed to extract clinical diseases, medications, and lab tests;
2. Assertion classification task, in which the previous track’s identified concepts are classified as being diagnosis or condition being present, absent, or possible, etc.;
3. The concept relation classification task is the classification of relationships between concepts into types. For example, clinical diseases may refer to tests in different ways such as “test reveals clinical condition”, “test performed to explore clinical condition”, or “even if it’s in the same sentence, the relationship is other/unknown”. For the 2010 shared task, 871 medical records were annotated.

The 2012 temporal relations shared task^52^ focused on temporal relationships in clinical notes. Two tracks participated in this shared task: 1) identification of clinical events and their occurrence times, and 2) identification of time and the temporal order of events. For the 2012 shared task, 310 clinical records were annotated. There are three shared tasks for the 2013 ShARe/CLEF eHealth Evaluation Lab^53^ which were information retrieval for medical queries, identification and normalization of diseases, and identification and normalization of abbreviations. The ShARe corpus of clinical records were used for the first two tasks, and more clinical data was augmented with those data for the third task.

**Table 1:** Some of the previous i2b2 challenge tasks involving identifying risk factors for heart disease in clinical notes.

Shared task (Year)	Objectives	Best evaluation (F-measre)	References
i2b2 de-identification and smoking challenge (2006)	Automatic identification of patient smoking status and de-identification of personal health information	De-identification: 0.98; Smoking identification: 0.90	^49, 54^
i2b2 obesity challenge (2008)	Identification of obesity and its co-morbidities	0.9773	^50^
i2b2 medication challenge (2009)	Identification of medications, their dosages, administration methods, frequencies, durations, and administration reasons from discharge summaries	Durations identification:0.525; Reason identification:0.459	^48^
i2b2 relations challenge (2010)	Concept extraction, and classification of assertion and relation	Concept extraction: 0.852; Classification of assertion and relation: 0.936	^51^
i2b2 coreference challenge (2011)	Coreference resolution	0.827	^55^
i2b2 temporal relations challenge (2012)	Extraction of temporal relations from clinical records involving identification of temporal expressions, temporal relations, and significant clinical events	Event: 0.92; Temporal expression: 0.90; Temporal relation: 0.69	^52^
i2b2 de-identification and heart disease risk factors challenge (2014)	Automatic de-identification and identification of CAD risk factors in the narratives of diabetes patients’ longitudinal clinical records	De-identification: 0.9586; Risk factor: 0.9276	^56, 57^
CLEF eHealth shared task 1 (2013)	Named entity recognition in clinical notes	0.75	^58^
CLEF eHealth shared task 1b (2014)	Normalization of abbreviations or acronyms	Task 2a: 0.868 (accuracy); Task 2b: 0.576 (F-measure)	^59^
CLEF eHealth shared Evaluation (2020)	Clinical named entity recognition from French clinical notes	Recognition of plain entity: 0.756; Recognition of normalized entity: 0.711; Entity normalization: 0.872	^60^
CLEF eHealth shared Evaluation (2021)	Clinical named entity recognition from French medical text	Recognition of plain entity: 0.702; Recognition of normalized entity: 0.529; j Entity normalization: 0.524	^61^
SemEval task 9 (2013)	Extraction of drug-drug interactions from clincial texts	Drugs recognition: 0.715; Drug-drug interactions extraction: 0.651	^62^
SemEval task 7 (2014)	Identification and normalization of diseases and disorders in clinical notes	Identification: 0.813; Normalization: 0.741 (accuracy)	^63^
SemEval task 14 (2015)	Named entity recognition and filling template slot for clinical notes	Named entity recognition: 0.757; Template slot filling accuracy:0.886; Recognition of disorder and template slot filling accuracy: 0.808	^64^
SemEval task (2016)	Extraction of temporal information from clinical notes involving identification of time expression, event expression and temporal relation	Identification of time expression: 0.795; Identification of event expression: 0.903; Identification of temporal relation: 0.573	^46^

## Material and methods

### Dataset description

The proposed model used a dataset provided from Partners HealthCare [http://www.partners.orghttps://www.i2b2.org/NLP/HeartDisease/] that contains clinical notes, and discharge summaries. The dataset provided for the 2014 i2b2/UTHealth shared task contains 1,304 clinical records describing 296 diabetes patients for heart disease risk factors and time attributes related to the DCT. The challenge provider divided the dataset into the training set that contains 60% of the total dataset (790 records), while the test set contains the other 40%. (514 records). The annotation guidelines define a set of annotations for identifying the existence of diseases (such as CAD, heart disease, and diabetes), relevant eight evidence risk factors (such as hypertension, hyperlipidemia, smoking status, obesity, and family history), and associated medications. Each risk factor category has its own set of indicators for detecting whether the disease or risk factor is present in the patient with the occurrence time (before, during, or after) the DCT.

Each heart disease risk factor has a time attribute that describes the relationship between the risk factor and the corresponding DCT. This relationship is similar to the temporal relationship between a clinical event and DCT in the 2012 i2b2 clinical NLP challenge^52^, except that the value of the time attribute can be any combination of “before”, “during”, or “after” rather than just a single variable consisting of “before”, “during,” and “after”. Most of participating systems in the 2012 i2b2 clinical NLP challenge have applied machine learning techniques to extract relationships between events and DCT^65, 66^. For example, Tang et al. developed the best system by using SVMs^65^.

More specifically, The annotators generated document-level tags for each heart disease risk factor indicator to identify the risk factor and its indicator existence of that patient, as well as whether the indicator was present before, during, or after the DCT. The i2b2 challenge annotation guideline^10^ provided more description details of patient risk factors with associated indicators.

**Figure 1:**
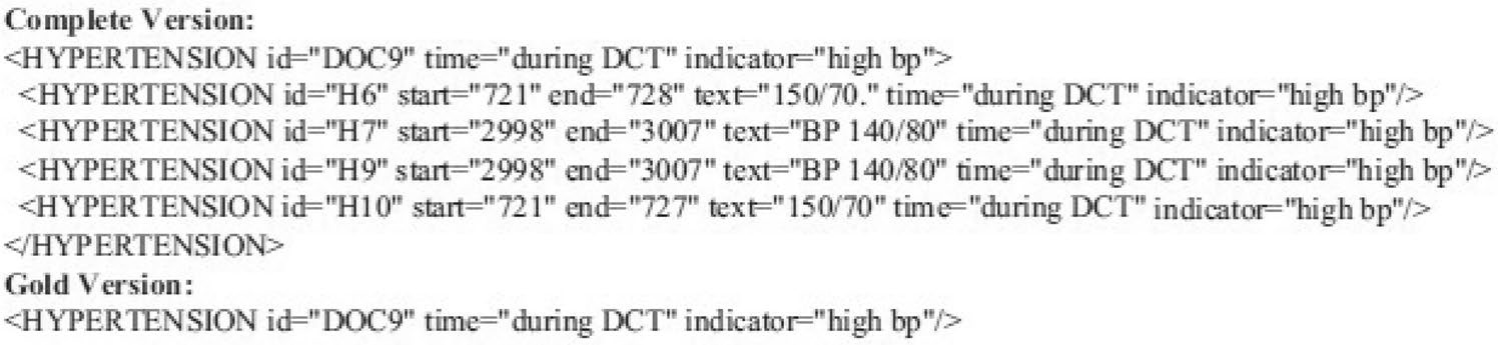
Example 1 of heart disease risk factors tags.

**Figure 2:**
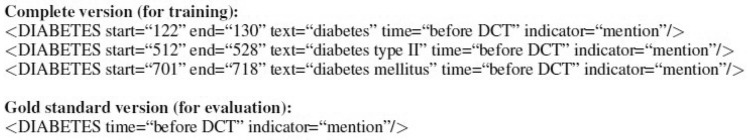
Example 2 of heart disease risk factors tags.

An example of the annotation tags used for the training and evaluation process is shown in Figs. 1 and 2 that are generated using MAE (Multi-purpose Annotation Environment)^67^. While the complete annotations contain token-level information (risk factor tags, risk factor indicators, offsets, text information, and time attributes), the gold standard annotations contain document-level information (risk factor tags, risk factor indicators, and time attributes) that cannot be duplicated.

Table 2 provides a brief description of the heart risk factors and their indicators as illustrated in^10^.

**Table 2:** An overview of each risk factor tag used in the shared task dataset.

Risk factor tags	Indicator	Time attribute	Number	
			Traning data	Testing data
(a) Tag: CAD Indicator	Mention, event, test, symptom	Time	1186	784
(b) Tag: DIABETES Indicator	Mention, high A1C, high glucose	Time	1695	1180
(c) Tag: HYPERLIPIDEMIA Indicator	Mention, high cholesterol, high LDL	Time	1062	751
(d) Tag: HYPERTENSION Indicator	Mention, high blood pressure	Time	1926	1293
(e) Tag: OBESE Indicator	Mention, high BMI	Time	433	262
(f) Tag: MEDICATION Type (type1)	ACE inhibitor, amylin, anti-diabetes, ARB, aspirin, beta-blocker, calcium channel blocker, diuretic, DPP4 inhibitors, ezetimibe, fibrate, GLP1 agonist, insulin, meglitinide, metformin, niacin, nitrate, obesity medications, statin, sulfonylurea, thiazolidinedione, thienopyridine	Time	8638	5674
(g) Tag: SMOKER Status	Current, past, ever, never, unknown	NA	771	512
(h) Tag: FAMILY_HIST Indicator	Present Not present	NA	790	514

The number of training and testing sets at the annotation level, and the indicators related to each risk factor for heart disease detection.

According to Chen et al.(2015)’s terminology, evidence of heart disease risk factor indicators may be divided into three categories as shown in Table 3:

1. Phrase-based indicators where the evidence is presented directly in sentences, such as “hyperlipidemia” or the name of a particular medication.
2. Logic-based indicators where the evidence is presented directly in sentences but required more logical inferences, such as finding a blood pressure reading and comparing the results to see if they are high enough to be considered as a risk factor.
3. Discourse-based indicators where the evidence is not presented directly, but are hidden in clinical notes and may require a parsing process, such as identifying smoking status or family history.

Sentence boundary identification and tokenization were the first tasks of the preprocessing module completed after receiving a raw data file including clinical text. Then the three tag extraction modules determined the type and indicator of the tags by extracting evidence of them from the three categories in Table 3. The time attribute identification module then identified the time attribute for each evidence item (if any exists). Finally, the evaluation module is performed after converting the complete version’s tags to the gold version’s tags. We applied the MedEx^68^ tokenization module, a medical information extraction tool, for sentence boundary recognition and tokenization. Then we developed an ensemble of Conditional Random Fields (CRF) and Structural Support Vector Machines (SSVMs)^69^ to identify phrase-based risk factors. For logic-based risk factors, we used rules and output from NegEx^70^, and discourse-based risk factors were identified by studying Support Vector Machines (SVMs). Finally, we assigned temporal features to risk factors using a multi-label classification approach. The phrase-based indicators extraction can be identified by matching medical keywords using named entity recognition (NER). Each token of evidence was identified by a BIOES tag, where S indicates the evidence token itself and B, I, O, and E indicate that the token is located at the beginning, middle, outside, or end of the token of evidence, respectively. As an example of evidence from the phrase-based tag in Table 3, the sentence “Continue beta blocker, CCB” was labeled as “Continue/O; beta/B-medication beta + blockers; blocker/E-medication_beta + blockers; ,/O; CCB/S-medication calcium-channel + blockers”, where “medication” is a type of tag and {“beta blockers”, “calcium-channel blockers”} are two indicators of this type of tag. The logic-based indicators extraction can be identified by interpreting the vital signs or measurements. There are two factors for extracting logic-based indicators which are:

- Identifying all numerical evidence, such as “LDL measurement of over 100 mg/dL”, which demonstrates the evidence of hyperlipidemia with high LDL as determined by
  $$LDL > 100\, \textrm{mg}/\textrm{dL}$$.
- Identifying all co-occurrence evidence by discovering all evidence based on several keywords, such as “Early-onset CAD in mother”, which is evidence of family history like “early, CAD, mother”. The only evidence of family history tags was extracted using this criterion.

The discourse-based indicators extraction. Unlike the other two tag categories discussed above, discourse-based tags do not explicitly state the evidence they include, making it challenging to directly extract it. In this model, we first developed evidence-candidate sentences with discourse-based tags based on indicator-related words or phrases, such as symptom-related phrases like “unstable angina,” and then we used SVMs to assess whether or not those sentences were indicators-related. The classifier used a variety of features, such as term frequency-inverse document frequency (TF-IDF) of words, unigrams, bigrams, negation information of sentences stated in the phrase-based tag extraction module, and negation information of indicator-related words/phrases identified by NegEx.

**Table 3:** Types of heart disease risk factor indicators evidences.

Evidence category	Risk factor indicator	Example
Phrase-based indicators	Medication	Important PMH for CAD, HTN, GERD, and previous cerebral embolism. Continue beta blocker , CCB.
Logic-based indicators	high bp, hypertension	BP 170/80 and was last seen in a local cardiac rehab centre. P 72, weight 276 lb, and BP 140/80.
Discourse-based indicators	CAD, event	Findings that indicate to a left circumflex distribution obstructive coronary lesion. His LAD stent was still in place after catheterization, although there was a 90% lesion next to the stent.

Based on the associated evidence and identified by its indicator(s), each tag described in Table 4 may fall under more than one of the categories mentioned above. The Table 4 shows the relationships between the tag categories and the tag types where each item indicates the category that a tag with an indicator belongs.

**Table 4:** Relationships between the risk factor tags and evidence category and the training set percentage for each type.

Risk factor tag	Phrase-based	Logic-based	Discourse-based
CAD	Mention	NA	Event, test result, symptom
Diabetes	Mention	High glucose, high A1c	NA
Hyperlipidemia	Mention	high LDL, high cholesterol	NA
Hypertension	Mention	High blood pressure	NA
Obesity status	Mention	Waist circumference, BMI	NA
Family history	NA	Present, not present	NA
Smoking status	NA	NA	All statuses
Medication	All types	NA	NA
Training set percentage	85.33	8.10	6.57

### Task description

Risk factors and temporal indicators were classified as a document-level classification task. This is a multilabel classification task, in which multiple labels are identified for a particular EHR. However, because of the unique nature of the annotation guideline^10^ and the structure of the training data, which includes phrase-level risk factors and time indicator annotations as shown in Figure 2, it recommends designing the problem as an information extraction task. Data is viewed as a sequence of tokens labeled using the Inside-Outside (IO) method in this method: Named entity tokens are indicated by I, while non-entity tokens are indicated by O. The major goal is to identify the risk factor indicators contained within the record, as well as the temporal categories of those indicators related to the DCT. Each entity is assigned a label in the following format:

I-risk_factor.indicator.time

Table 5 shows an example of an EHR that is represented by a sequence of terms and their labels. In this instance, the label “I-cad.mention.before_dct” with the word “CAD” with can be considered as a mention of CAD that occurred before the DCT.

**Table 5:** A sample phrase in an EHR and their labels.

Words	she, has, CAD, and, hypertension she, has, coronary, artery, disease, and, diabetes
Labels	O, O, I-cad.mention.before_dct, O, I-hypertension.mention.before_dct O, O, I-cad.mention.before dct, I-cad.mention.before dct, I-cad.mention.before dct, O, I-diabetes.mention.before dct

### Clinical word embeddings

#### General contextual embeddings

Word embeddings are the basis of deep learning for NLP. Traditional word-level vector representations, such as word2vec^71^, GloVe^72^, and fastText^73^, demonstrate all possible word meanings as a single vector representation and are unable to distinguish BERT^74^ has proposed contributions in the recent years by generating contextualized word representations. ELMo can be applied to several NLP tasks as a language model to generate a context-sensitive embedding for each word in a phrase by pre-training on a large text dataset. BERT is deeper and has many more parameters than ELMo, giving it a powerful representation. Instead of just providing word embeddings as features, BERT can be applied to a downstream task and optimized as a task-specific architecture. BERT has been demonstrated to be significantly more effective than non-contextual embeddings in general and ELMo in particular on several tasks, including those in the clinical domain^30^. As a result, we will apply BERT in this paper, instead of ELMo or other non-contextual embedding techniques.

#### Contextual clinical embeddings

There are several studies have proposed and applied contextual models in clinical and biomedical applications. BioBERT^29^ uses PubMed [https://www.ncbi.nlm.nih.gov/pubmed/] article abstracts and PubMed Central [https://www.ncbi.nlm.nih.gov/pmc/] article full texts to train a BERT model across a corpus of biomedical research publications.

They observe that the structure provided by clinical texts converted to better performance on a variety of clinical NLP tasks, and they released their pre-trained BERT model. Regarding clinical text^75^, apply a general-domain pre-trained ELMo model to de-identify clinical text, reporting near-state-of-the-art performance on the i2b2 2014 challenge^10, 57^ and on several aspects of the HIPAA PHI dataset.

Two studies use the clinical dataset to train contextual embedding algorithms. The first study proposed by^76^ improved performance on the i2b2 2010 task by training an ELMo model using a clinical dataset of discharge summaries, radiology notes, and medically relevant Wikipedia articles^51^. Along with their research, they provide a pre-trained ELMo model, allowing future clinical NLP research to use these powerful contextual embeddings. The second one was published by^30^ in 2019 providing promising results on all four corpora which are the i2b2 2010 and 2012 tasks^52, 77^ and the SemEval 2014 task 7^63^ and 2015 task 14^64^ tasks by training a clinical note corpus BERT language model and using complex task-specific models to outperform both conventional embeddings and ELMo embeddings.

### Ethical approval

This article does not contain any studies with human participants or animals performed by any of the authors.

## The proposed heart disease risk factors detection model

In this section, we provide a detailed description of the developed model to extract risk factors of heart disease from clinical notes over time using the 2014 i2b2 clinical NLP challenge dataset. These risk indicators were extracted initially, and then their time aspects were identified. In this section, we present the proposed model steps by explaining preprocessing steps, describing the pre-trained word embeddings, and stacked word embeddings.

- The proposed model applies BERT and CharacterBERT independently on the given document which contains clinical notes.
- After embedding the words and before inputting representations into the document RNN, the hidden size is 512 and the reprojected word dimension is 256, creating a fully connected layer.
- Then merge the vectors of all BERT’s subword embeddings of the same word (e.g. by averaging them) to word embedding and concatenate it to CharacterBERT embeddings.
- The document embedding is generated by concatenating BERT embedding of size 768-length embedding vector and Character-BERT embedding of size 768-length vector embeddings.
- Once we have the clinical note embeddings, a classification model can use the generated vectors as input to predict heart disease risk factors. With model interpretability in mind, we used RNN to predict heart disease risk factors in the IO format.

### Motivations

Every day, avoidable heart attacks cause needless deaths. Doctors’ and clinicians’ notes from routine health care visits provide all the disease risk factors. In this research, we show how advanced NLP and Deep Learning approaches may be used to interpret these notes and turn them into useful insights. This research shows how machine learning and artificial intelligence have advanced in their ability to process and interpret unstructured text data.

### The proposed models

The proposed model detected each type of tag in the following order:

- First, extract evidence (if any exists) by type and indicator.
- Then, Determine the attribute (i.e., time, if it exists).

For example, the case of hypertension with a “mention” indicates a phrase-based tag, while a case of hypertension associated with another indicator indicates a logic-based tag, as observed in the example from Figure 1. The training set contains 85.33%, 8.10%, and 6.57%, respectively, of phrase-, logic-, and discourse-based tags as detailed in Table 4. The training set contains 85.33%, 8.10%, and 6.57%, respectively, of phrase-, logic-, and discourse-based tags. After all tags have been assigned to the three categories in Table 3, we applied a unified framework for each category. Figure 5 shows an overview of the proposed model which is divided into the following modules: a preprocessing module that extracts three tags and identifies the time attribute, then a stacked Word embeddings module and a post-processing module.

### Preprocessing

Preprocessing steps involve concept mapping and sentence splitting. Metamap^78^ was applied to map the words and phrases in the clinical notes to concepts. Meanwhile, for sentence splitting, we used Splitta^79^ which is an open-source machine-learning-based tool. Once a word or phrase has been mapped to the concepts we’re concerned with (for example, family group, disease or syndrome, smoke, etc.), the sentence it belongs to will be identified as one of the candidate sentences to be processed further. The target concepts are determined when Metamap is used to process the annotation set.

#### Pre-trained language models

This section briefly described the most common available feature vectors known as the pre-trained embeddings which were used in this study.

#### BERT model

Devlin et al.^74^ has an important impact on the improvement of NLP domain. BERT language model is trained to predict the masked words in a text for many languages by combining the Wikipedia corpora. This model is fine-tuned and applied to various monolingual and multilingual NLP tasks with limited data. BERT is ground-breaking since it successfully outperformed the results for major NLP tasks. BERT sparked as much excitement in the NLP community as ImageNet did for computer vision. This is what we intended to do using clinical text data to extract risk factors for a disease. We used BERT as a classifier and as an embedding in our NLP/Deep Learning models to show the potential of BERT. The process of converting text data into vectors is called embedding. The main benefit of employing BERT was its capacity to comprehend a word’s context due to the bidirectional nature of the embedding itself. Transformators process input sequences simultaneously, in contrast to conventional RNNs. They extract the relationships between words in an input sequence and store its order using self-attention and positional embeddings.

#### CharacterBERT

**Figure 3:**
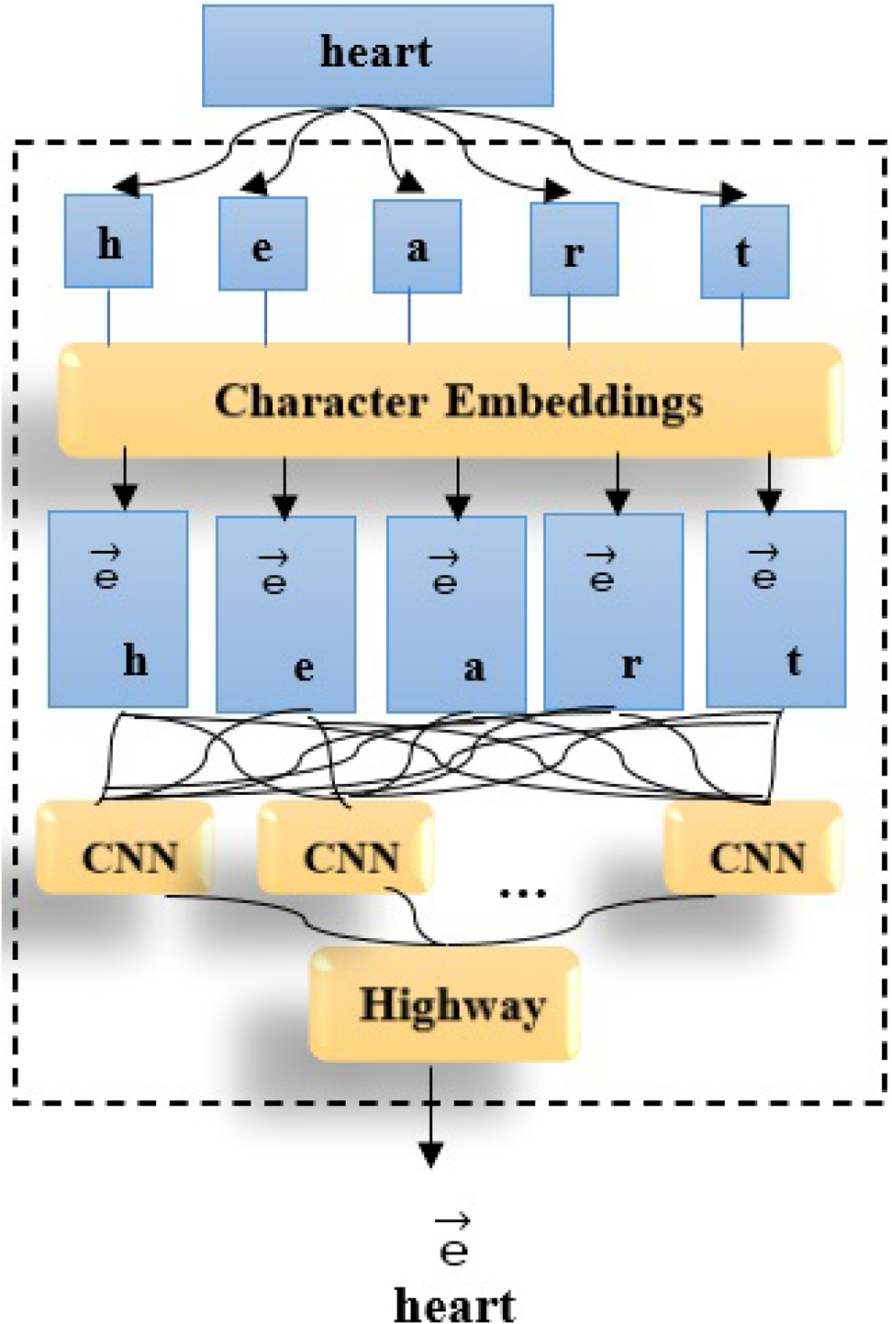
CharacterBERT-based embedding methodology.

Boukkouri et al.^80^ is a BERT variation that generates word-level contextual representations by focusing on each input token’s characters. CharacterBERT employs a CharacterCNN module, which is similar to ELMo^81^, to generate representations for arbitrary tokens instead of depending on a matrix of pre-defined word pieces. Besides this difference, CharacterBERT has the same architecture as BERT. The CharacterBERTmedical model is derived from CharacterBERTgeneral retrained on a medical corpus. Character-CNN represents BERTmedical in Character-CNN form. In BERT, token embeddings were produced as single embeddings. The CharacterBERT module uses the CharacterCNN module instead of WordPieces embedding, which is very important when working in specialized fields such as the clinical domain. Consequently, CharacterBERT can handle any input token as long as it is not excessively long (i.e. less than 50 characters). Following that, a character embedding matrix is used to represent each character, producing a sequence of character embeddings. Then this sequence is passed to multiple CNNs which process the sequence n-characters at a time. The outputs from each CNN are combined into a single vector, which is then mapped using Highway Layers to the required dimension^82^ as shown in Figure 3. The context-free representation of the token is contained in this final vector, which will be merged with position and segment embeddings before being passed to several Transformer Layers as in BERT. BERT’s vocabulary is not appropriate for phrases with specific terms (for example, “choledocholithiasis” is divided into [cho, led, och, oli, thi, asi, s]). While the clinical wordpiece performs better, it still has some limitations (for example, “borborygmi” becomes “bor, bor, yg, mi”). Thus, a BERT version called CharacterBERT was developed to avoid any inefficiencies that may result from using the incorrect WordPiece vocabulary. Clinical CharacterBERT appears to be a more reliable model than clinical BERT.

#### Flair

Akbik et al.^19^ is a language model used to generate contextual word embeddings. Despite being the same character string, words can be interpreted differently by models because words are contextualized by the text around them. In our research, we applied the multi-forward and multi-backward model, where forward and backward refer to the traversal direction of word in a phrase. It was trained in over 300 languages on the JW300 corpus.

#### Recurrent neural network (RNN)

**Figure 4:**
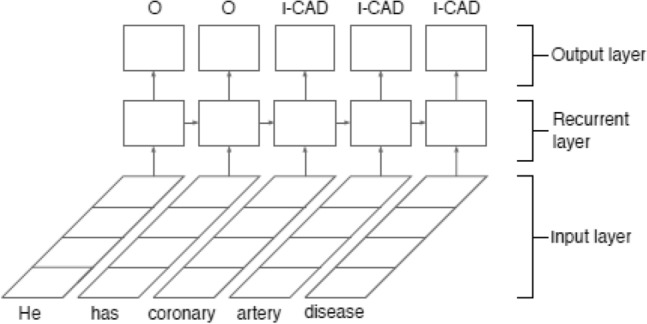
RNN structure for heart disease risk factors detection.

Once we have the clinical note embeddings, a classification model can use the vectors as input to predict the diagnostic code. With model interpretability in mind, we used a recurrent neural network (RNN) to predict heart disease risk factors. A recurrent neural network is a type of neural network that is designed to analyze sequential data. Unlike CNN, the RNN learns the representation of clinical text using a recurrent layer, as shown in Figure 4. The entire clinical document is represented by a word sequence of length *l* that is fed into an RNN using a matrix. $\textrm{S}\in \mathbb {R}^{d*l}$:

$$\begin{aligned} \textrm{S} =\left[ W_{1} W_{2}\ldots W_{l}\right] \end{aligned}$$

where $\textrm{W}_{i} \in \mathbb {R}^{d}$ is the ith word’s representation as a d-dimensional word vector in S. A hidden state output *hi* is generated in an Elman-type network^83^ by the nonlinear transformation of an input vector *Wi* and the previous hidden state $h_{i-1}$.

$$\begin{aligned} h_{i} =f (h_{i-1},W_{i}) \end{aligned}$$

where *f* is a recurrent unit, such as a GRU, and LSTM. Finally, to detect a risk factor in the IO format, the hidden state $h_{i}$ is fed into softmax.

#### Stacked word embeddings

**Figure 5:**
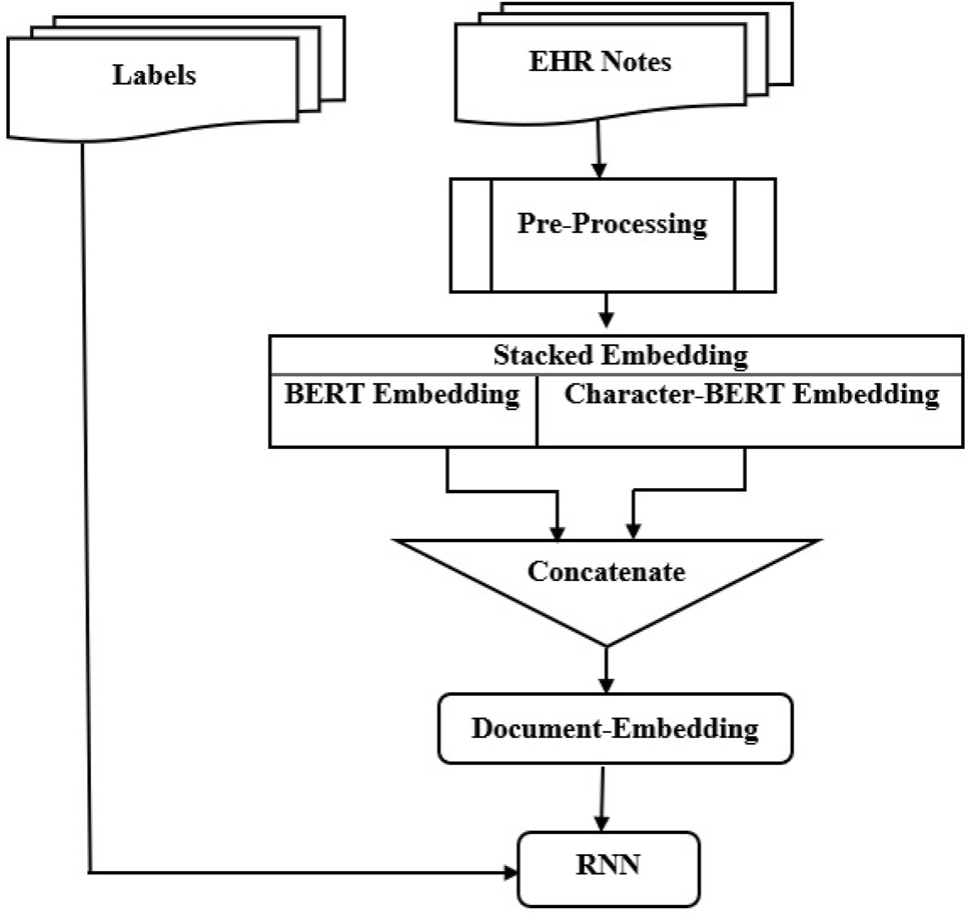
The proposed stacked word embeddings model.

**Figure 6:**
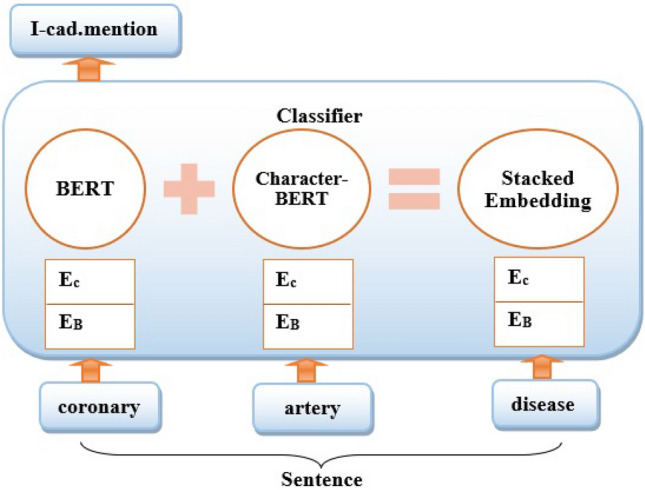
Stacked embeddings where EB is (BERT Embedding) and EC is (CharacterBERT embedding).

According to the previous study^84^, stacking multiple pre-trained embeddings provides higher performances than employing only a one-word embedding technique. Stacking is the process of combining the final feature vectors from multiple language models to form a single feature vector with more textual features as shown in Figure 5. For classification tasks, stacking is an efficient ensemble learning technique because it combines multiple base classification models via a meta-classifier. We employed stacked embeddings, which included BERT with CharacterBERT and an RNN classifier on top of these stacked embeddings. We developed a number of models using BERT, including token classifiers, sentence classifiers, and ensemble models. Also, we developed a powerful technique of stacking embeddings, as shown in the Figure 6 which demonstrates how stacked embeddings generate a new embedding for the given document that is the input for the RNN to predict heart disease risk factors. We proposed a new technique based on stacking token embeddings from the BERT and Character-BERT models by concatenating their results and generating new token embeddings to get the best performance and improved robustness to misspellings. The new embedding length is the result of adding the length of BERT and Character-BERT embeddings. The proposed model uses the Document-Embeddings over the word stack so that the classifier can identify how to combine the embeddings for the classification task. Document embedding is initialized by passing a list of word embeddings that are BERT embedding and Character-BERT embedding. Then DocumentRNNEmbeddings will be used to train an RNN on them. The RNN takes the word embeddings of every token in the document as input and outputs the document embeddings as its last output state. RNN can categorize the patient according to risk factors for heart disease based on the particular characteristics of the annotation and the structure of the training data, which includes phrase-level risk factors and time indicator annotations.

## Experimental results and simulations

In this section, we provide a detailed description of the developed model results that achieves the best result compared to state-of-the-art models from the 2014 i2b2/UTHealth shared task as listed in Table 6.

**Table 6:** Experimental results of proposed model and previous systems from 2014 i2b2/UTHealth shared task.

Model	Recall	Precision	F1-score
Proposed model	0.9265	0.9366	**0.9366**
Roberts et al.^36^	**0.9625**	0.8951	**0.9276**
Chen et al.^85^	0.9436	** 0.9106**	0.9268
Cormack et al.^86^	0.9375	0.8975	0.9171
Yang and Garibaldi^1^	0.9488	0.8847	0.9156
Shivade et al.^87^	0.9261	0.8907	0.9081
Chang et al.^88^	0.9387	0.8594	0.8973
Khalifa and Meystre^89^	0.8951	0.8552	0.8747
Karystianis et al.^90^	0.9007	0.8557	0.8776
Chokkwijitkul et al.^7^	0.9180	0.8983	0.9081

Significant values are in [bold].

The proposed model has significant improvement as a universal classifier since it provides 93.66% in F-measure when compared to the top-ranked systems^36, 85, 88^ which use a hybrid of knowledge-and data-driven techniques, and systems^86, 89, 90^ that only use knowledge-driven techniques, such as lexicon and rule-based classifiers.

### Evaluation metrics

The result of a given EHR is a sequence of tags, each tag corresponding to a single word. The final result, after deleting duplicate tags, the record will have a set of unique tags (excluding the O label). The output for the example in Table 5 will ultimately consist of two distinct labels, containing “I-cad.mention.before_dct” and “I-hypertension.mention.before_dct”. With the use of these labels, system annotations such as that in Figure 2 will be generated, the proposed model was evaluated using the evaluation script provided by the challenge organizers that outputs macro-/micro-precision, - recall, and -F1-score, of which micro-precision and -F1-score were used as the primary measurements [The official evaluation script is available at https://github.com/kotfic/i2b2_evaluation_scripts].

### Discussion

The model generated an overall microaveraged F1-measure of 93.6%, a macro-averaged F1-measure of 70% and weighted-avg F1-measure of 96% as shown in Table 7. The overall results that are macro- and weighted-averaged, as well as the macro-averaged analysis of the results for each class of heart disease provided in terms of Precision, Recall, and F1-measure are shown in Table 8 and Table 9.

**Table 7:** The overall results of the proposed model at the heart risk indicator level.

Risk factor	Precision	Recall	F1-score	Support
Other	0.98	0.99	**0.98**	38,375
Smoker	0.70	0.60	0.65	457
Diabetes	0.79	0.69	0.74	582
Obese	0.00	0.00	0.00	116
Cad	0.87	0.56	0.68	446
Family_hist	0.87	0.90	**0.88**	13
Hypertension	0.92	0.85	**0.88**	664
Hyperlipidemia	0.82	0.92	0.87	231
Medication	0.82	0.51	0.63	2062
Accuracy			**0.96**	42,946
Macro avg	0.75	0.67	0.70	42,946
Weighted avg	0.96	0.96	0.96	42,946

Significant values are in [bold].

**Table 8:** The overall results that are macro- and weighted-averaged, as well as the macro-averaged analysis of the results for each class of information provided in terms of Precision, Recall, and F1-measure.

Risk factor	Indicator	Precision	Recall	F1-score	Support
Diabetes	a1c	0.67	0.92	0.77	64
	Glucose	1.00	0.07	0.13	29
	Mention	0.98	1.00	0.99	489
CAD	Event	0.71	0.76	0.74	173
	Mention	0.84	0.93	0.88	183
	Symptom	0.89	0.80	0.84	65
	Test	0.89	0.12	0.21	25
Hypertension	High_bp	0.98	0.99	0.98	186
	mention	1.00	0.99	0.99	478
Hyperlipidemia	High_chol	0.00	0.00	0.00	7
	High_ldl	0.85	0.74	0.79	24
	Mention	0.98	1.00	0.99	200
OBESE	Obese_bmi	0.00	0.00	0.00	9
	Mention	0.93	1.00	0.97	107
Smoker	Smoker_current	0.00	0.00	0.00	36
	Smoker_ever	0.00	0.00	0.00	3
	Smoker_never	0.93	0.96	0.94	111
	Smoker_past	0.78	0.85	0.81	110
	Smoker_unknown	0.99	0.97	0.98	197
Medication		0.82	0.51	0.63	2062
Family history		0.87	0.90	0.88	13
Accuracy				0.9366	42,946
Macro average		0.3383	0.2920	0.2899	42,946
Weighted avg		0.9265	0.9366	0.9290	42,946
Micro average				0.9366	42,946

**Table 9:** The overall results that are macro- and weighted-averaged, as well as the macro-averaged analysis of the results for each class provided with time-attribute provided in terms of Precision, Recall, and F1-measure.

Risk indicator	Time attribute	Precision	Recall	F1-score	Support
Diabetes	Before_dct	0.78	0.85	0.81	278
	During_dct	0.49	0.33	0.39	204
	After_dct	0.00	0.00	0.00	100
CAD	After_dct	0.67	0.63	0.65	107
	Before_dct	0.78	0.93	0.85	258
	During_dct	0.00	0.00	0.00	81
Hypertension	After_dct	0.89	0.79	0.84	116
	Before_dct	0.00	0.00	0.00	53
	During_dct	0.79	0.87	0.83	495
Hyperlipidemia	After_dct	0.00	0.00	0.00	97
	Before_dct	0.00	0.00	0.00	107
	During_dct	0.66	0.95	0.78	27
OBESE	After_dct	0.73	0.67	0.70	15
	Before_dct	0.00	0.00	0.00	41
	During_dct	0.89	0.75	0.82	60
Medication	After_dct	0.61	0.26	0.36	706
	Before_dct	0.62	0.42	0.50	798
	During_dct	0.67	0.34	0.45	558
Accuracy				0.9366	42946
Macro average		0.3383	0.2920	0.2899	42946
Weighted avg		0.9265	0.9366	0.9290	42946
Micro average				0.9366	42946

For CAD, Diabetes, Hyperlipidemia, Hypertension, and family history of CAD, the best accuracy for indicators of disease, with micro averaged F1-measures of 98%, 99%, 1.00%, 99%, and 94.94%, respectively. The accuracy of identifying medications, obesity mentions, and smoking status was 85.85%, 86.12%, and 86.55%, respectively, using micro-averaged F1 measures. On an overall basis, a significant performance is achieved by stacking embeddings and RNN as a classifier over these stacked embeddings. The results achieved the best improvement by using stack of different word embeddings instead of using only one word embedding.

**Figure 7:**
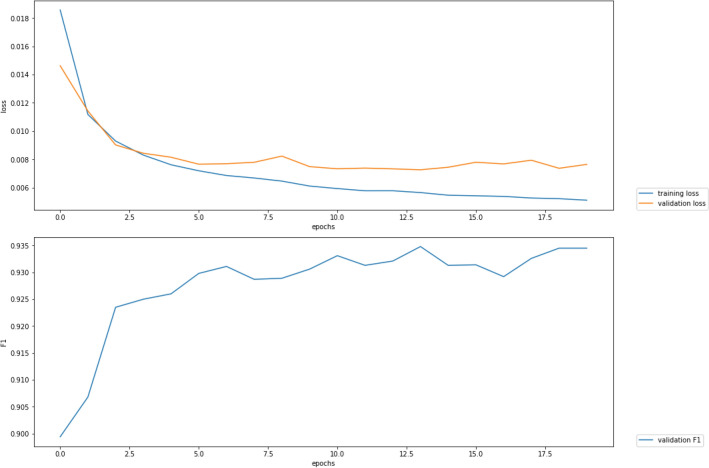
F1-plot curve of train and validation learning.

Stacking BERT and CharacterBERT embeddings provides a promising result, which is 93.66% micro averaged F1-measures. All approaches demonstrate a significant performance of combining BERT and CharacterBERT embeddings. The BERT-CharacterBERT model outperforms the med-bert and biobert embeddings in case of a single type of pre-trained embeddings for classification, respectively as shown in Table 10. A significant performance is achieved by stacking embeddings compared to those with Flair backward and forward. Figure 7 show F1-Plot.

**Table 10:** All experiments have been evaluated on the test set.

Model type	F1-score (%)
microsoft (med-bert)	91
biobert (https://github.com/dmis-lab/biobert/)+characterBert	92.7
bertConfig+CharacterBert	**93.66**
bertConfig+CharacterBert+focalLS	93.45
microsoft+focalLs	91.05
microsoft+characterBert	91.28

Significant values are in [bold].

Using the 2014 i2b2 clinical NLP dataset, we developed a model to detect heart disease risk factors, and medications from clinical notes over time based on DCT. Evaluation of the proposed model achieved significant results with the highest F1-score of 93.66%. It should be mentioned that, while using stacked word embeddings, the proposed model’s performance was comparable to that of the system with the highest performance. We used the i2b2 shared task dataset, which included clinical text data that have been annotated by humans. We investigated employing BERT as both a classifier and a dynamic (contextual) embedding under the assumption that embedding has a significant impact on the performance of the model. The data was given in XML format with annotations, as seen in the example above 1. The BERT+Character stacking embedding model outperformed all the other models we tested. We identified predictions that were accurate and overlooked by human annotators by analyzing the outcomes from our models. The results also showed how effective contextual embeddings are. Based on the context in which the relevant text appeared, it was possible to detect risk factors.

### Error analysis

As previously mentioned, the prediction process of the heart disease risk indicators involved three steps: First, the occurrences of relevant evidence are detected in the text; Second, the relevant time attribute tag is assigned to each identified evidence (except for FAMILY HIST and SMOKER). The results of the evidence detection and temporal attribute identification are then combined to develop a set of risk factor annotations. Here, we categorize model errors into two groups: evidence-level errors, which include the evidence occurrences that are incorrectly identified or that are missing, and time-attribute errors, which include occurrences of risk indicators that are correctly identified but are assigned the incorrect time attribute.

1. Evidence-level errors
  There are five major categories to classify evidence-level errors: (1) In certain circumstances, the overall contexts must be taken into account when identifying special terms. For example, in specific cases, the terms ‘CAD’ and ‘coronary artery disease’ are only labeled as the [CAD: mention] indicator. (2) The model can not identify token-level of previously unobserved evidence on the test data (such as ‘ischemic cardiomyopathy’ and ‘Acute coronary syndrome’). (3) The tags SMOKER STATUS and FAMILY_HIST were incorrectly categorized. For example, The misclassification of ’previous’ and ’unknown’ into the ’present’ tag causes quite a few false positives in the SMOKER tag. (4) The small training data and complex contexts are the main factors behind the majority of false positives or negatives for the errors in terms of sentence-level clinical facts. (5) For clinical assessments at the sentence level, simple and well-presented indicators (such as ‘A1C’, ‘BMI’, and ‘high bp’) provide better results than complex indicators, such as ‘glucose’ and ‘high chol.’, which are needed when taking into account.
  Table 7 indicates that our model performs well ($F1 > 0.8$) in extraction for four risk factors (diabetes, family history, hyperlipidemia, and hypertension). The confusion matrix shows that the “Other” class is far more frequently confused with the (CAD, diabetes, hypertension, and hyperlipidemia) classes than the other (CAD, diabetes, hypertension, and hyperlipidemia) classes. Despite our data augmentation, there is still an imbalance in the classes between the “Other” and “CAD, diabetes, hypertension, and hyperlipidemia” classes. The confusion matrices for the previous mentioned tags’ indicators are shown in Tables 11, 12, 13, 14.
2. Time-attribute errors
  The completeness and efficiency of the developed model are major factors of well-time-attribute annotations. However, the model was unable to develop precise heuristics to capture the properties of these time attribute tags because some time attribute tags had insufficient training instances, such as the after DCT tag regarding the [CAD:event] and [CAD:symptom] indicators, which had fewer than 10 instances. The confusion matrices for time attribute of the previous tags’ indicators are shown in Tables 15, 16, 17, and 18. These matrices show that a lot of the mentioned tags classes have been confused with “Other” class in the prediction with the examples as shown in Table 19 and 20.

**Table 11:** Confusion matrix for error analysis for CAD tag indicators predictions.

	pred:Other	pred:event	pred:mention	pred:symptom	pred:test
true:Other	20,432	81	38	68	23
true:event	87	166	35	4	4
true:mention	25	19	246	3	0
true:symptom	60	1	3	49	0
true:test	37	3	7	2	20

**Table 12:** Confusion matrix for error analysis for diabetes tag indicators predictions.

	pred:A1C	pred:Other	pred:glucose	pred:mention
true:A1C	47	47	0	14
true:Other	21	34,497	4	120
true:glucose	0	39	4	1
true:mention	2	60	0	717

**Table 13:** Confusion matrix for error analysis for hyperlipidemia tag indicators predictions.

	pred:Other	pred:high LDL	pred:high chol	pred:mention
true:Other	24,858	6	1	34
true:high LDL	16	16	0	1
true:high chol.	5	0	1	1
true:mention	31	0	0	311

**Table 14:** Confusion matrix for error analysis for hypertension indicators tag predictions.

	pred:Other	pred:high bp	pred:mention
true:Other	36,573	59	69
true:high bp	26	187	3
true:mention	32	4	685

**Table 15:** Confusion matrix for error analysis for CAD tag time predictions.

	pred:Other	pred:after DCT	pred:before DCT	pred:during DCT
true:Other	20,455	81	38	68
true:after DCT	6	6	56	0
true:before DCT	193	169	199	39
true:during DCT	34	14	36	19

**Table 16:** Confusion matrix for error analysis for diabetes tag time predictions.

	pred:Other	pred:after DCT	pred:before DCT	pred:during DCT
true:Other	34,503	40	45	54
true:after DCT	15	101	46	42
true:before DCT	61	13	118	22
true:during DCT	52	124	84	236

**Table 17:** Confusion matrix for error analysis for hyperlipidemia tag time predictions.

	pred:Other	pred:after DCT	pred:before DCT	pred:during DCT
true:Other	24,832	25	22	20
true:after DCT	13	15	15	7
true:before DCT	31	26	60	37
true:during DCT	7	7	26	138

**Table 18:** Confusion matrix for error analysis for hypertension time tag predictions.

	pred:Other	pred:after DCT	pred:before DCT	pred:during DCT
true:Other	36,576	35	34	56
true:after DCT	4	115	16	10
true:before DCT	16	182	23	166
true:during DCT	34	30	140	201

**Table 19:** Sample from dataframe generated from error analysis for CAD tag indicators predictions.

SentenceID	Sentence	Label	File	Class0	Class1	Class2	Class3	Class4	predClass	predLabel
66	70 yo M with multiple cardiac risk factors and.	Symptom	110-03.xml	0.000793	0.000240	0.000303	0.998302	0.000362	Class3	Symptom
86	71 yo M with CAD, s/p CABG x 4 in 3/80.	Event	110-04.xml	0.000804	0.993561	0.004396	0.000401	0.000837	Class1	Event
98	Coronary artery disease : s/p CABG x .	Event	110-04.xml	0.001814	0.003055	0.994300	0.000270	0.000561	Class2	Mention
157	Sternal pain– non-exertional, reproducible by.	Event	110-04.xml	0.001314	0.996738	0.000688	0.000601	0.000660	Class1	Event
161	Pericarditis a possibility (he had post-op per.	Event	110-04.xml	0.001491	0.996681	0.000750	0.000558	0.000520	Class1	Event
180	65-year-old male with known history of CAD who.	Mention	111-04.xml	0.002081	0.000973	0.996085	0.000404	0.000457	Class2	Mention
192	PAST MEDICAL HISTORY: Hypertension, diabetes,.	Mention	111-04.xml	0.002119	0.000964	0.996061	0.000422	0.000434	Class2	Mention
251	Prior to his pacemaker placement, an exercise .	Other	112-03.xml	0.397554	0.004942	0.000649	0.587603	0.009252	Class3	Symptom
253	The test was terminated for 7/10 substernal ch.	Test	112-03.xml	0.000901	0.000225	0.000318	0.998172	0.000384	Class3	Symptom
289	He complained of fatigue and exertional throat.	Test	112-04.xml	0.000908	0.000529	0.000285	0.000495	0.997784	Class4	Test
290	Cardiac catheterization performed by Dr. Lesli.	Test	112-04.xml	0.053236	0.008662	0.000666	0.001351	0.936086	Class4	Test
291	He received a 3 mm stent, postdilated to 3.5 mm,.	Event	112-04.xml	0.001716	0.996366	0.000925	0.000404	0.000590	Class1	Event

**Table 20:** Sample from dataframe generated from CAD tag time predictions.

SentenceID	Sentence	Label	File	Class0	Class1	Class2	Class3	predClass	predLabel
8	HPI: 70 yo M with NIDDM admitted for cath aft.	Before DCT	110-03.xml	0.999235	0.000022	0.000710	0.000033	Class0	Other
12	MIBI was read as positive for moderate to seve.	Before DCT	110-03.xml	0.995930	0.000060	0.003940	0.000071	Class0	Other
60	The ECG is positive for ischemia.	Before DCT	110-03.xml	0.985374	0.000127	0.014360	0.000139	Class0	Other
62	Findings are consistent with moderate to sever.	Before DCT	110-03.xml	0.999231	0.000032	0.000698	0.000039	Class0	Other
68	\tIschemia: Hx angina, MIBI positive for infer.	Before DCT	110-03.xml	0.999664	0.000042	0.000254	0.000040	Class0	Other
94	The pain does not remind him of his sx prior t.	Before DCT	110-04.xml	0.804428	0.000567	0.194260	0.000744	Class0	Other
182	walking, took 2 nitro and the pain got better.	Before DCT	111-04.xml	0.999683	0.000022	0.000273	0.000022	Class0	Other
184	repeat episode relived by nitro again.	Before DCT	111-04.xml	0.999844	0.000016	0.000124	0.000016	Class0	Other
198	PAST SURGICAL HISTORY: Angioplasty with multi.	Before DCT	111-04.xml	0.802693	0.000435	0.196281	0.000591	Class0	Other
257	He tells me that he underwent testing at Wheat.	Before DCT	112-03.xml	0.997462	0.000051	0.002432	0.000056	Class0	Other

## Conclusion and future work

In this research, we developed a clinical narratives model for identifying heart disease risk factors that can detect diseases, associated risk factors, associated medications, and the time they are presented. The proposed model has used stacked word embeddings which have demonstrated promising performance by stacking BERT and CHARACTER-BERT embedding on the i2b2 heart disease risk factors challenge dataset. Our method achieved F1-score of 93.66%, which provides significant results compared to the best systems for detecting the heart disease risk factors from EHRs. Our work also demonstrates how contextual embeddings may be used to increase the effectiveness of deep learning and natural language processing. This research work is a start toward an implementation that, with just minor feature engineering changes, might outperform the current state-of-the-art results and develop a system that can perform better than human annotators. One of the future directions is to involve more modern approaches such as deep learning and ensemble learning to deal with the complicated risk factors.

## Data Availability

The datasets provided during the current study are available: http://www.partners.org and https://www.i2b2.org/NLP/HeartDisease/.
